# Plastome Evolution in Hemiparasitic Mistletoes

**DOI:** 10.1093/gbe/evv165

**Published:** 2015-08-29

**Authors:** Gitte Petersen, Argelia Cuenca, Ole Seberg

**Affiliations:** Natural History Museum of Denmark, University of Copenhagen, Denmark

**Keywords:** hemiparasite, *Viscum*, gene loss, pseudogenes, structural rearrangements, selection, intergenic regions

## Abstract

Santalales is an order of plants consisting almost entirely of parasites. Some, such as *Osyris*, are facultative root parasites whereas others, such as *Viscum*, are obligate stem parasitic mistletoes. Here, we report the complete plastome sequences of one species of *Osyris* and three species of *Viscum*, and we investigate the evolutionary aspects of structural changes and changes in gene content in relation to parasitism. Compared with typical angiosperms plastomes, the four Santalales plastomes are all reduced in size (10–22% compared with *Vitis*), and they have experienced rearrangements, mostly but not exclusively in the border areas of the inverted repeats. Additionally, a number of protein-coding genes (*matK*, *infA*, *ccsA*, *rpl33*, and all 11 *ndh* genes) as well as two transfer RNA genes (*trnG-UCC* and *trnV-UAC*) have been pseudogenized or completely lost. Most of the remaining plastid genes have a significantly changed selection pattern compared with other dicots, and the relaxed selection of photosynthesis genes is noteworthy. Although gene loss obviously reduces plastome size, intergenic regions were also shortened. As plastome modifications are generally most prominent in *Viscum*, they are most likely correlated with the increased nutritional dependence on the host compared with *Osyris*.

## Introduction

Parasitic plants receive water and nutrients through haustorial connection with host plants. They may be obligate parasites depending on host connection throughout their entire life-time, or they may be facultative parasites being able to live part of their life independent of a host. Several obligate parasites have lost photosynthetic activity entirely (holoparasites), whereas others have retained photosynthesis (hemiparasites). Loss of photosynthetic activity is assumed to relax normal constraints on evolution of the chloroplast genome (plastome), and a number of studies on the evolution of the plastome in parasitic plants have been made (e.g., Funk et al. 2007; McNeal et al. 2007; Braukmann et al. 2013; Wicke et al. 2013). Complete plastome sequences are now available for a number of species representing different types of parasitism in Orobanchaceae (Wolfe et al. 1992; Li et al. 2013; Wicke et al. 2013) and *Cuscuta* L. (Convolvulaceae) (Funk et al. 2007; McNeal et al. 2007).

Another evolutionary pathway to an achlorophyllous lifestyle is mycoheterotrophy. Mycoheterotrophic plants are also connected to a host plant, but through a mycorrhizal fungus as an intermediate. The loss of photosynthesis in mycoheterotrophs is assumed to have a similar impact on their plastomes as on those of holoparasites, and several complete plastomes of mycoheterotrophic plants have now been sequenced. These plastomes are mostly from orchids (Delannoy et al. 2011; Logacheva et al. 2011; Barrett and Davis 2012; Barrett et al. 2014; Schelkunov et al. 2015), as well as two nonorchid monocotyledon mycoheterotrophs, *Petrosavia stellaris* Becc. (Logacheva et al. 2014) and *Sciaphila densiflora* Schltr. (Lam et al. 2015).

As expected plastomes degrade with decreasing dependence on photosynthesis (Funk et al. 2007; McNeal et al. 2007; Wicke et al. 2013). Most notably in the holoparasite *Rafflesia lagascae* Blanco the plastome may have been lost completely (Molina et al. 2014). Plastomes have been found in all other holoparasites and mycoheterotrophs investigated so far, though in the mycoheterotroph orchid *Epipogium roseum* (D.Don) Lindl. the plastome is reduced in size to approximately 19 kb (Schelkunov et al. 2015). Apart from major deletions of both normally coding and noncoding regions, general modifications of the plastomes of parasitic and mycoheterotrophic plants include pseudogenization, structural rearrangements, and possibly horizontal gene transfer.

The currently available data from different groups of plants showing different types of parasitism or mycoheteretrophy permit comparative studies aimed at discovering general pathways of plastome degradation (Krause 2011; Barrett and Davis 2012; Wicke et al 2013; Barrett et al. 2014). However, with parasitism being shown to have evolved 12 times within the angiosperms (Westwood et al. 2010; Su et al. 2015) and mycoheterotrophy at least 50 times (Merckx and Freudenstein 2010), the current data cover only a small fraction of the actual diversity. Thus, to fully understand the evolutionary pathways associated with parasitism and mycoheterotrophy plastome data from many more groups of plants are needed.

One group, hitherto not investigated in terms of complete plastome evolution, is the order Santalales, which is composed mostly of hemiparasites, but includes also autotrophs confined to three small families (Erythropalaceae, Strombosiaceae, and Coulaceae) and holoparasites in Balanophoraceae and Mystropetalaceae. With more than 2,300 species this is the largest group of parasitic plants, comprising almost half of all known parasitic plants (see Parasitic Plant Connection at http://www.parasiticplants.siu.edu/ParPlantNumbers.pdf, last accessed September 2, 2015). Phylogenetic analyses of Santalales have provided much insight into the evolution of parasitism, although some relationships are still unclear (Vidal-Russell and Nickrent 2008; Nickrent et al. 2010; Su et al. 2015). Thus, hemiparasitism may have evolved only once but possibly twice, root parasitism is the ancestral state with stem parasitism having evolved five times independently, and holoparasitism has surprisingly evolved twice. All hemiparasitic species are capable of photosynthesis, but in some stem parasites levels of photosynthesis are reduced (Tuquet and Sallé 1996; Strong et al. 2000; Logan et al. 2002). The photosynthetic tissue of a few stem parasitic species is also strongly reduced. In *Viscum minimum* Harv. (Viscaceae), *Arceuthobium* M.Bieb. spp. (Viscaceae) and *Tristerix aphyllus* (DC) Barlow & Wiens (Loranthaceae) the main vegetative body grows endophytically within the host, and it has been suggested that such species may represent an evolutionary road toward holoparasitism (Heide-Jørgensen 2008; Nickrent and García 2009).

**Fig. 1.—:**
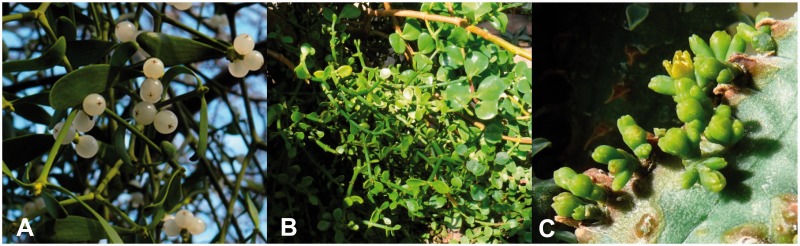
The three species of *Viscum* studied. (*A*) *Viscum album* with white, ripe fruits. (*B*) *Viscum crassulae* growing on *Portulacaria afra*. (*C*) Flowering *V. minimum* emerging from the stem of the succulent *Euphorbia mammillaris*.

Including all life forms from autrophy to hemiparasitism and holoparasitism Santalales is an obvious candidate for studies of plastome evolution. In a pilot study we here describe and compare complete plastomes from the root parasite, *Osyris alba* L. (Santalaceae) that appears to be facultative (Nickrent 2002) and three obligate stem parasite species of *Viscum* L. (fig. 1): *Viscum album* L., *Viscum crassulae* Eckl. & Zeyh., and *V. minimum*. We ask the following questions: To what extent does hemiparasitism influence plastome evolution? Has the almost entirely endophytic nature of *V. minimum* led to a higher level of plastome degradation compared with other *Viscum* species with a larger body of green, exophytic tissue? Does hemiparasitism influence selection pressure on plastid genes? Although no other complete Santalales plastomes are currently available, sequence data from the inverted repeat (IR) region of *Heisteria concinna* Standl. (nonparasite, Erythropalaceae), *Ximenia americana* L. (facultative root hemiparasite, Ximeniaceae), and *Phoradendron leucarpum* (Raf.) Reveal & M.C.Johnst. (obligate stem hemiparasite, Viscaceae) (Moore et al. 2011) are available and provide still further insight into the evolution of that particular region.

## Materials and Methods

### Plant Material, DNA Extraction, and Sequencing

Plant samples were collected from specimens grown in the Botanical Garden, Natural History Museum of Denmark. Voucher specimens are deposited in the herbarium (C) of the Natural History Museum of Denmark: *Osyris alba* (C2883), *V. album* (C2546), *V. crassulae* (C2553), and *V. minimum* (C2884). For some comparisons with other Santalales sequences, the IR regions from *H. concinna* (HQ664616), *X. americana* (HQ664594), and *P. leucarpum* (HQ664580) (Moore et al. 2011) were used.

Green leaves were used for DNA extraction of *Osyris alba*, *V. album* and *V. crassulae*, whereas green seeds were used for DNA extraction of the minute *V. minimum*. In all cases, approximately 100 mg of fresh tissue was used for total genomic DNA extractions using a standard CTAB method (Doyle JJ and Doyle JL 1987). Illumina short-insert, paired-end (PE) libraries with average insert size of 500 bp were constructed for *V. minimum*, *V. crassulae* and *Osyris alba,* and each sample was run in 1/16 of a lane on an Illumina HiSeq 2500. For *V. album*, single-read libraries with an average insert size of 400 bp were constructed and run in a whole lane on an Illumina HiSeq 2500. Librairies and sequencing data were produced at the Danish National High-Throughput DNA Sequencing Centre.

To complete plastid genome assembly and verify an inversion in *V. minimum*, polymerase chain reaction (PCR) was performed using primer pairs Viscum_F1 (5′-CTGTTACTGTTATAGACTGTCTG-3′)/Viscum_R1 (5′-GCACGTTGCTTTCTACCACATCG-3′) and Viscum_F2 (5′-CTGTTCAAATTTCATCTCTATCG-3′)/Viscum_R2 (5′-GCGTAGGCCTCAGGCAATTTGGC-3′). PCR products were purified using the QIAquick PCR purification kit (QIAGEN). Cycle sequencing was performed using the ABI PRISM Dye Terminator Cycle Ready Reaction kit with AmpliTaq DNA Polymerase, FS (Applied Biosystems, Wellesley, MA), and the products were purified as above. Sequencing was conducted on an ABI 3130XL automated sequencer (Applied Biosystems).

### Genome Assembly, Annotation, and Analysis

All sequence read processing and plastome assembly were performed using Geneious v.6-7 (Biomatters, Inc., Auckland, New Zealand). Low-quality 5′- and 3′-ends (error probability limit 0.05) were trimmed of both PE and single read sequences. Trimmed reads of *V. minimum* were mapped to two reference plastomes of *Vitis vinifera* (NC_007957) and *Fagopyrum esculentum* (NC_010776). Reads were mapped with medium-low sensitivity and two iterations. Mapped reads were extracted from the references and de novo assembled using the Geneious assembler at medium-low sensitivity. Consensus sequences of contigs were extended by using them as references for five times iterative mapping of all trimmed reads. New consensus sequences were generated and used for de novo assembly. This procedure was iterated until one large contig was obtained. A single gap needed to be closed by PCR followed by Sanger sequencing using primers designed to match flanking regions (see above). For assembly of the *Osyris* and other *Viscum* plastomes, the same procedure was used except that the *V. minimum* plastome was also used as a more accurate initial reference sequence.

Annotation of the *Osyris* plastome was conducted in DOGMA (Wyman et al. 2004), which uses BLASTX searches against a database of annotated plastid genomes and allows for manual determination of start/stop codons. Additional comparisons to annotated plastomes in GenBank, primarily from species of *Vitis* such as *Vitis rotundifolia* Michx. (NC_023790), were used to guide the determination of coding regions and exon/intron boundaries. The transfer RNAs (tRNAs) without intron were annotated using tRNAscan-SE (Schattner et al. 2005), those with introns by comparison to *Vitis* sequences in GenBank. The *Viscum* plastomes were manually annotated in Geneious v.6-7 by comparison to *Osyris* and *Vitis* plastomes.

The amount of repetitive DNA sequences was determined using the REPuter program (Kurtz et al. 2001). Forward and palindromic (reverse complement) repeat longer than 20 bp and with an *E* value > 0.1 was counted applying a Hamming distance of 3.

### Test for Relaxed Selection

Individual genes were aligned in Geneious v.8 (Biomatters) using the option Translation Align, which translates DNA sequences into amino acid sequences, and subsequently does the alignment using MUSCLE with default settings. Some gene matrices were concatenated into gene groups (*atp*, *pet*, *psa*, *psb*, *rpo*, *rpl*, *rps*, and *ycf3+4*), whereas others were treated individually (*accD*, *cemA*, *clpP*, *rbcL*, and *ycf2*). The *ycf1* gene was not used due to extensive alignment ambiguity.

Tests for potential-relaxed selection of protein-coding genes in parasitic species of Santalales were performed using the hypothesis testing framework RELAX (Wertheim et al. 2015) available on the Datamonkey webserver at www.datamonkey.org/RELAX (last accessed September 2, 2015) as part of the HyPhy software package (Pond et al. 2005; Delport et al. 2010). Although other programs usually only test whether the ratio of nonsynonymous (d*N*) to synonymous (d*S*) substitutions, *ω*, deviates from the neutral expectation of *ω* = 1, RELAX calculates a selection intensity parameter, *k*, taking into account that relaxation will have different effect on sites subjected to purifying selection (*ω* < 1) and sites subjected to positive selection (*ω* > 1). Relaxation will move *ω* toward 1 for both categories. RELAX tests whether selection is relaxed or intensified on a subset of test branches compared with a subset of reference branches in a predefined tree. In the null model, the selection intensity is constrained to 1 for all branches, whereas in the alternative model *k* is allowed to differ between reference and test groups. Acceptance or rejection of the alternative model is tested using a likelihood-ratio test, but we also include values of the Akaike Information Criterion with a correction for finite sample size (AICc) as measures of fit of the null model and the alternative model, respectively. For comparison to other papers, we also calculate *ω* values from the partitioned MG94xREV model.

A constrained phylogenetic tree including *Viscum*, *Osyris,* and 14 representatives of autotrophic plants from other orders of dicotyledons (see supplementary table S1, Supplementary Material online) was constructed following the Angiosperm Phylogeny Group (APG III 2009) and using *Ranunculus* to place the root. In one set of analyses, we tested changes in selection in the parasitic species (test group) compared with 13 eudicots (reference group). We also tested whether there was any difference in the selection pattern between facultative and obligate parasites (*Osyris* vs. *Viscum*).

To increase taxon sampling in Santalales, another test was performed using the five protein-coding genes (*rpl2*, *rpl23*, *rps7*, *rps12*, and *ycf2*) located in the IR where sequences were available for three more species: One autotroph (*H. concinna*), one facultative parasite (*X. americana*), and one obligate parasite (*P. leucarpum*). Complete IR sequences were aligned using LASTZ ver. 1.02.00 allowing gaps (Harris 2007). As input to RELAX a phylogenetic tree was constructed by maximum likelihood using RAxML 7.2.8 (Stamatakis 2006) with the GTR+gamma model. Relaxed selection was tested for parasitic versus autotrophic species, obligate versus facultative parasites, and obligate parasitic versus facultative plus autotrophic species. As analyses based only on data from Santalales have low statistical power, another set of analyses was performed including eight other eudicots as reference (see supplementary table S1, Supplementary Material online).

## Results

### Genomic Data and Plastome Structure

The total number of Illumina reads (of untrimmed length 101 bp) and the coverage of each plastome are listed in table 1. Compared with the number of reads, the coverage of *V. minimum* is lower than that of the other *Viscum* plastomes. The difference is likely caused by the different tissues—seeds versus leaves—used for DNA extraction. The comparatively much higher coverage of the *Osyris* L. plastome may be caused by a favorable plastid to nuclear genome DNA ratio.

**Table 1:** GenBank Accession Numbers, the Number of Illumina Reads, and Mean Coverage of Each Plastome

Species	GenBank Accession number.	Reads No.	×-Coverage
*Osyris alba* L.	KT070882	7,273,026	432.7
*Viscum album* L.	KT003925	24,000,000^a^	145.2
*Viscum crassulae* Eckl. & Zeyh.	KT070881	24,000,000	158.2
*Viscum minimum* Harv.	KJ512176	40,000,000	156.2

^a^More than 200,000,000 reads were obtained, but only 24,000,000 were used here.

**Fig. 2.—:**
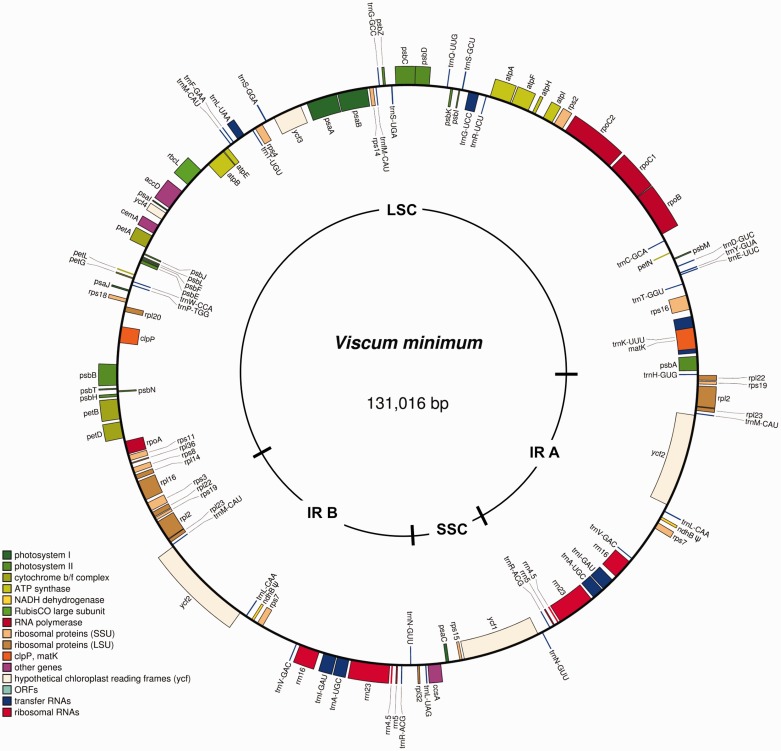
Circular map of the plastome of *V. minimum*. Genes shown outside the outer circle are transcribed clockwise and those inside are transcribed counterclockwise. Pseudogenes are marked by ψ. Drawing made using OGDRAW v.1.2 (Lohse et al. 2013).

The size of each complete plastome and other genome characteristics are listed in table 2 and the plastome of *V. minimum* is shown in figure 2. None of the nonparasitic members in Santalales was available for plastome sequencing, thus the *Vit. rotundifolia* plastome was selected for comparison as it had the highest similarity to the *Osyris* plastome based on a BLASTN search. The plastome of *Osyris* is the most complete of the four plastomes sequenced here being less than 10% reduced compared with *Vitis* L. *Viscum* plastomes are more reduced with *V. crassulae* being approximately 22% reduced compared with *Vitis*. In *Osyris* as well as in the *Viscum* species, the small single copy (SSC) region is relatively most reduced. Some size reduction is caused by deletion of genes, but intergenic regions are also affected. A list of the lengths of 80 intergenic regions located between functional, homologous genes shared by *Vitis*, *Osyris,* and *Viscum* reveals a general size reduction of 8% in *Osyris* compared with *Vitis* and reductions between 24% and 32% in the *Viscum* species (supplementary table S2, Supplementary Material online).

**Table 2:** Plastome Characteristics of Four Species of Santalales Compared with *Vitis*

	Vitis	Osyris	Viscum album	Viscum crassulae	Viscum minimum
Genome size	160,891	147,253 (91.5%)	128,921 (80.1%)	126,064 (78.4%)	131,016 (81.4%)
LSC	89,090	84,601 (95.0%)	73,893 (82.9%)	73,226 (82.2%)	75,814 (85.1%)
SSC	19,247	13,972 (72.6%)	8,632 (44.8%)	8,628 (44.8%)	9,014 (46.8%)
IR	26,277	24,340 (92.6%)	23,198 (88.3%)	22,105 (84.1%)	23,094 (87.9%)
GC%^a^	37.4	37.7	36.4	36.4	36.2
GC% in LSC	35.3	35.6	33.5	33.6	33.3
GC% in SSC	31.5	31.2	24.8	24.0	24.2
GC% in IR	43.0	43.1	43.2	43.4	43.2

Note.—Numbers in parentheses are sizes compared with *Vitis*.

^a^G+C base percentage of each plastome.

The length of the IR has been reduced approximately 12–16% in the *Viscum* species compared with *Vitis*; however, the borders are not the same. The LSC–IRb border of *Vitis* is in the 5′-end of *rps19*, but in *Osyris* it has shifted to the *rps19–rpl2* intergenic region. However, in *Viscum* it has been moved in the opposite direction being located either in the 3′-end of *rpl22* (in *V. minimum*) or to the *rpl22–rps3* intergenic region (*V. album* and *V. crassulae*). The IRa–LSC border in all plastomes except that of *V. album* is located downstream from the *trnH-GUG* gene, by convention located where plastome numbering begins. However, in *V. album* this gene is included in the IR region. In all *Viscum* species, the SSC/IR border is located in the *ycf1* gene with just 70–95 bp in the duplicated IR regions. In *Vitis,* the IR/SSC border is also located in the *ycf1* gene with 1,030 bp being duplicated. In *Osyris,* the IR is longer than in *Viscum* and reduced only approximately 7% compared with *Vitis*. However, an inversion (approximately 3,260 bp) has occurred in the SSC region, which affects the IRb/SSC border. The *rpl32*–*trnL*–*ccsA* region (normal orientation) has been inverted and the IRb/SSC border is located in *ccsA* with 149 bp being found also in the IRa region. Thus, no part of the *ycf1* gene is duplicated and the two tRNAs, *trnN* and *trnR* usually located within the IR are only present once in the 3′-end of the SSC.

A larger structural change was found within the large single copy (LSC) region of *V. minimum*, where an approximately 24,450-bp-long inversion has occurred. The borders of this inverted region are located between *rps16*/*trnQ-UUG* and *trnT-GGU*/*psbD* in the normally oriented plastomes.

**Fig. 3.—:**
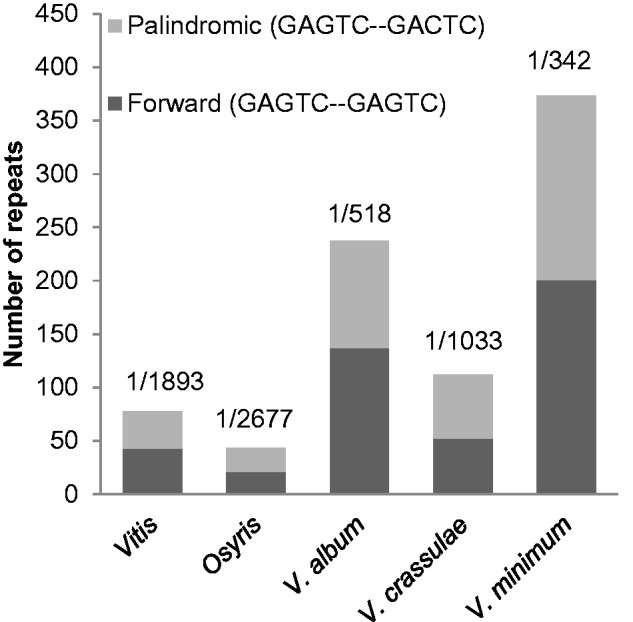
Proportions of palindromic and forward repeat DNA in plastomes of *Vitis* and four species of Santalales. Numbers above columns indicate the repeat density, for example, 1/1,000 means that a repeat occurs every 1,000 bp.

The amount of repetitive DNA varies greatly among species (fig. 3). *Vitis* has 85 repeats and *Osyris* only 55, whereas the three species of *Viscum* have 122–383 repeats. Except in *Vitis* (with 73%) more than 80% of the repeats are between 20 and 30 bp. The GC content of *Osyris* (37.7%) is slightly higher than in *Vitis* (37.4%), but in *Viscum* is has been reduced (36.2–36.4%) (table 2).

### Gene Content

The plastome of *Osyris* has the highest number of potentially functional genes with a total of 101 including 67 protein-coding genes, 4 ribosomal RNA (rRNA) genes, and 30 tRNA genes (supplementary table S3, Supplementary Material online). In *Viscum,* potentially functional gene numbers vary from 96 in *V. album* to 99 in *V. minimum*. Compared with *Osyris* all species of *Viscum* have lost the *trnV-UAC* gene and *V. album* and *V. crassulae* have also lost the *trnG-UCC* gene.

With respect to the protein-coding genes, all species of *Viscum* have lost one of the ribosomal protein-coding genes (*rpl33*) that is present in *Osyris*. In *Osyris,* a functional *rpl33* gene (204 bp) is located in the 816-bp-long intergenic region between *psaJ* and *rps18*. In the species of *Viscum,* this region is reduced to a length between 440 bp (in *V. crassulae*) and 529 bp (in *V. album*) without resemblance to *rpl33* gene sequences.

The species of *Viscum* also share the complete loss of the *infA* gene, which is present in *Osyris* as a pseudogene. A substitution at position 10 downstream from the start codon creates a stop codon. Further downstream two length mutations (compared with *Vit. rotundifolia*), a 4-bp deletion and a 1-bp insertion, can be observed. The *infA* pseudogene (231 bp) in *Osyris* is located in the 479-bp-long region between *rpl36* and *rps8*. This region is reduced in *Viscum* to a length between 204 bp (in *V. album*) to 382 bp (in *V. crassulae*) without resemblance to *infA* gene sequences.

In *V. album,* both *ccsA* and *matK* have been pseudogenized. In the *matK* gene, a 2-bp deletion some 270 bp downstream from the start codon interrupts the reading frame. In the *ccsA* gene, approximately 520 bp downstream from the start codon one or more deletions totaling 61 bp compared with *V. minimum* interrupt the reading frame.

All four species share the functional loss of the whole suite of *ndh* genes, although they differ in the number and length of pseudogenes or still recognizable gene sequence fragments. In *Osyris,* shorter or longer fragments of eight *ndh* genes can be found. Five *ndh* pseudogenes (*ndhB*, *ndhC*, *ndhD*, *ndhE,* and *ndhK*) are recognizable over their entire length, including the *ndhB* intron. Most of *ndhH* can be recognized, but only fragments of about half the size of normal *ndhA* and *ndhG* genes can be identified. The 520-bp-long pseudogene sequence of *ndhA* is composed of parts of both the exons, but the intron sequence is entirely missing. The *ndhF*, *ndhI,* and *ndhJ* genes are completely missing.

The most conserved *ndh* gene sequence in *Osyris* is the *ndhB* pseudogene sequence showing 95% similarity to several eudicot sequences. The remaining *ndh* pseudogenes in *Osyris* show 74–84% similarity to the best matching sequence in GenBank. Despite being a pseudogene, *ndhB* has not been reduced in length compared with the functional *ndhB* gene in *Heisteria* (table 3). Neither has the pseudogenized *ndhB* sequence in *Ximenia*. In *Viscum*, the only *ndh* gene remaining is *ndhB*, but only shorter fragments can be recognized. In *V. album* and *V. minimum ndhB* is represented by fragments of 361 and 363 bp, respectively, and in *V. crassulae* by a shorter 192 bp fragment. The *V. album* and *V. minimum* sequences are both composed of two parts being similar to the same short regions of exon 1 and exon 2, but the intron is entirely lost. The *V. crassulae* fragment includes even shorter regions of the exon 1 and exon 2 sequences present in the two other species, and in addition a very short region similar to the *Osyris ndhB* intron. In *Phoradendron,* a still shorter fragment of only 143 bp remains. This region is similar to part of the exon 1 sequences found in the longer fragments of *Viscum*.

**Table 3:** Size of *ndhB* Genes, Pseudogenes, and Fragments in Seven Species of Santalales

*ndhB*	Exon1	Intron	Exon2
***Heisteria concinna***	777	678	762
*Ximenia americana*	781	683	754
*Osyris alba*	777	697	774
*Viscum album*	210	—	151
*Viscum minimum*	202	—	161
*Viscum crassulae*	40	51	101
*Phoradendron leucarpum*	143	—	—

Note.—Taxon in bold has a functional *ndhB* gene.

### Selection in Plastid Genes

In general, we found no significant differences between selection patterns of *Osyris* and *Viscum* (data not shown), so all parasitic plants were treated as a homogenous test group. There is strong evidence that selection of all photosynthesis genes of *Viscum* and *Osyris* is relaxed (*k* < 1) (table 4). Some of the other genes show the same pattern (*rpo* and *rps* genes), whereas the pool of *rpl* genes does not change significantly, and four genes, *accD*, *cemA*, *clpP* and *ycf2*, show increased selection intensity (*k* > 1).

**Table 4:** Test for Relaxed Selection of Genes in Four Parasitic Species Compared with a Dicot Reference Group

Gene or Gene Group	Relaxation Coefficient (*k*)	*P* Value	Likelihood Ratio (LR)	ω^a^	ω^a^	AICc	AICc
				(Reference)	(Parasites)	Null	Alternative
Photosynthesis genes							
*atp*	0.74	0.0003	13.26	0.097	0.149	43,791.15	43,777.67
*pet*	0.33	0.0000	31.72	0.063	0.158	19,082.06	19,052.36
*psa*	0.31	0.0000	32.33	0.045	0.104	35,687.49	35,657.16
*psb*	0.87	0.0013	10.27	0.051	0.098	47,893.82	47,885.55
*rbcL*	0.00	0.0001	15.08	0.126	0.050	12,203.80	12,185.51
*ycf3+4*	0.63	0.0009	10.96	0.150	0.303	9,631.26	9,622.34
Concatenated	0.77	0.0000	45.41	0.127	0.191	215,811.91	215,768.50
Other genes							
*accD*	1.35	0.0069	7.30	0.454	0.583	18,567.94	18,562.66
*cemA*	4.41	0.0352	4.43	0.427	0.404	8,052.09	8,049.72
*clpP*	6.06	0.0107	6.51	0.310	0.149	5,593.79	5,589.35
*rpl*	0.98	0.8755	0.02	0.209	0.266	20,315.29	20,317.28
*rpo*	0.80	0.0000	24.91	0.232	0.353	104,719.36	104,696.45
*rps*	0.64	0.0000	33.93	0.196	0.366	43,755.08	43,723.17
*ycf2*	1.15	0.0147	5.96	0.858	0.960	53,461.20	53,457.25
All concatenated	0.85	0.0000	25.89	0.162	0.232	261,368.65	261,344.76

Note.—The relaxation coefficient, *k*, is the estimated selection intensity.

^a^Calculated under the partitioned MG94xREV model.

The effect on selection of evolution from autotrophy (*Heisteria*) to facultative parasitism (*Ximenia* and *Osyris*) and finally to obligate parasitism (*Viscum* and *Phoradendron*) in Santalales was analyzed using five individual genes from the IR, but most tests revealed insignificant changes in selection (table 5). Only the long *ycf2* gene showed a highly significant increase in selection intensity associated with the evolution of obligate parasitism. Combined analyses of all genes confirmed increased selection to be associated with this evolutionary shift, but also for the evolution of parasitism in general. Statistic significance of the latter result was weak when only one autotroph (*Heisteria*) was included in the analysis, but it was increased when additional autotrophic eudicots were included.

**Table 5:** Test for Relaxed Selection of IR Genes in Seven Species of Santalales

Gene	Test and Reference Branches	Relaxation Coefficient (*k*)	*P* Value	Likelihood Ratio (LR)	AICc Null	AICc Alternative
*rpl2*	Obligate (4) versus other Santalales (3)	1.18	0.6533	0.20	2,973.30	2,975.17
	Obligate (4) versus facultative (2)	3.53	0.2776	1.18	2,987.23	2,988.13
	Parasitic (6) versus nonparasitic (1)	0.88	0.8305	0.05	2,972.70	2,989.33
*rpl23*	Obligate (4) versus other Santalales (3)	1.34	0.5754	0.31	1,192.24	1,194.13
	Obligate (4) versus facultative (2)	0.84	0.7473	0.10	1,200.95	1,203.09
	Parasitic (6) versus nonparasitic (1)	11.77	0.0382	4.30	1,192.24	1,190.14
*rps7*	Obligate (4) versus other Santalales (3)	1.28	0.8426	0.04	1,686.88	1,688.98
	Obligate (4) versus facultative (2)	1.20	0.8922	0.02	1,699.77	1,701.92
	Parasitic (6) versus nonparasitic (1)	0.97	1.0000	0.00	1,686.88	1,689.02
*rps12*	Obligate (4) versus other Santalales (3)	0.38	0.2730	1.20	892.37	893.40
	Obligate (4) versus facultative (2)	0.38	0.3065	1.05	905.74	906.97
	Parasitic (6) versus nonparasitic (1)	0.61	0.7092	0.14	892.37	894.46
*ycf2*	Obligate (4) versus other Santalales (3)	3.43	0.0000	21.93	29,701.22	29,681.30
	Obligate (4) versus facultative (2)	3.42	0.0000	17.87	29,700.87	29,680.73
	Parasitic (6) versus nonparasitic (1)	1.86	0.0657	3.39	29,699.85	29,698.47
Concatenated	Obligate (4) versus other Santalales (3)	3.91	0.0000	25.31	35,643.12	35,623.89
	Obligate (4) versus facultative (2)	3.11	0.0000	20.26	35,644.29	35,626.04
	Parasitic (6) versus nonparasitic (1)	2.18	0.0263	4.94	35,638.67	35,635.74
	Parasitic (6) versus nonparasitic (1 + 7)	2.13	0.0000	30.32	51,239.15	51,210.83

Note.—Numbers in parentheses are species with a given type of parasitism. The last comparison includes seven non-Santalales dicots.

## Discussion

To address whether characteristics of the plastomes of hemiparasitic Santalales have evolved as a response to parasitism, one should ideally include comparisons to plastomes from autotroph species of the same order; however, such sequences are currently not available. As explained above we have chosen to base comparisons on the plastome of *Vitis* as an alternative.

*Osyris alba* is a facultative hemiparasite being able to survive for years without host contact (Nickrent 2002), thus its plastome cannot be expected to differ significantly from those of autotrophic plants. Compared with *Vitis* and autotrophic angiosperms in general the plastome of *Osyris* is however, slightly reduced in size (table 1). Size reduction is most notable in the SSC region primarily due to the reduction or deletion of the seven *ndh* genes located in this region (see below). All species of *Viscum* are obligate hemiparasites, but they are photosynthetic and their slow growing seedlings may survive for months before establishing contact with their hosts (Visser 1981; Zuber 2004). The plastomes of the three species of *Viscum* investigated here are, however, modified more than in *Osyris* as their total size has been reduced to some 20% compared with *Vitis*. The primary reduction is again confined to the SSC region, where all the *ndh* genes have been deleted completely. The major part of the IR region has previously been sequenced in the autotroph *Heisteria* and the facultative hemiparasitic *Ximenia* (Moore et al. 2011). These sequences do not differ significantly in length from the comparable region (approximately 25 kb) of *Vitis*, thus, suggesting that subsequent size reductions, not just of the IR region but of the plastome in general, may be a response to increased nutritional dependence on the host.

The majority of previous studies of plastomes from parasitic or mycoheterotrophic plants has focused on nonphotosynthetic species, thus comparisons between the hemiparasites of Santalales and those of other group will be limited. In Orobanchaceae, the obligate hemiparasite *Schwalbea* L. has a plastome slightly larger than the autotrophic *Lindenbergia* Link & Otto despite the fact that a few genes have become pseudogenized (Wicke et al. 2013). Species of *Cuscuta* are usually considered obligate hemiparasites (Heide-Jørgensen 2008), but with very different levels of photosynthesis. Species with the most efficient photosynthesis have larger plastomes than those with less efficient photosynthesis, and a reduction (23–25%) has taken place compared with the plastome of the most closely related autotrophic species sequenced so far (Funk et al. 2007; McNeal et al. 2007). The most intact *Cuscuta* plastomes have experienced gene loss and pseudogenization comparable to the levels we have observed in *Viscum*, and it seems that overall size reduction is also at a similar level.

The overall reduction of plastome size cannot only be explained by loss of the *ndh* genes and the few other protein-coding genes deleted or pseudogenized (supplementary table S3, Supplementary Material online), but have involved intergenic regions as well (supplementary table S2, Supplementary Material online). Intergenic regions between functional homologous genes have been reduced by 8% in *Osyris* compared with *Vitis* and by 24–32% in the three species of *Viscum*. A similar observation was made by McNeal et al. (2007), showing that intergenic regions in two *Cuscuta* species decreased in size and that the decrease was most pronounced in the species with the least photosynthetic activity. Why these regions between apparently functional genes seem to shrink with increasing degrees of parasitism remains unknown. In the autotrophic species of Gnetales, size reduction of introns and intergenic spacer regions lead (Wu et al. 2009) to suggest that the smaller plastomes had evolved to facilitate a more economic use of resources under disadvantageous conditions.

Although the borders between the IRs and the single copy regions are mostly quite conserved among angiosperms, several alterations have been described in parasitic plants (Krause 2011). As described above, none of the borders is conserved among the plastomes of *Osyris* and *Viscum*. Changes of the LSC–IR borders in *Viscum* tend to increase the size of the IR compared with *Vitis*, but the SSC–IR borders move in the other direction, more or less compensating for the gains. Thus, the overall size reductions of approximately 12–16% of the *Viscum* plastomes are primarily caused by deletions of gene and intergenic sequences (table 2). In *Osyris*, changes to both LSC–IR and SSC–IR borders reduce the IR size. The change of the SSC–IR border to the *ccsA* gene, probably a result of inversion of the *rpl32*, *trnL* and *ccsA* gene region, is responsible for the entire approximately 7% size difference between the *Vitis* and *Osyris* IR regions. Whether any of the changes to the IR borders described here are actually related to the parasitic nature of these plants cannot be determined with the available evidence. Further data from species in the group as well as from autotrophic relatives are needed and the few available IR sequences from other members of the Santalales do not include border regions.

An inversion very similar to the one found in the SSC region in *Osyris* has been found in some species of *Cuscuta,* where the region including *ccsA* and *trnL-UAG*, but not *rpl32*, has been inverted (Funk et al. 2007; McNeal et al. 2007). The *ccsA–rpl32* region is flanked by *ndh* genes in normal plastomes, thus modifications to the region following pseudogenization and eventually complete gene loss are likely to facilitate structural changes. This possibility was also suggested by McNeal et al. (2007) although only one of borders of the *Cuscuta* inversion was flanked by an *ndh* gene. The current observation from *Osyris* with inversion borders normally being flanked by *ndh* genes fits the hypothesis even better. Another inversion observed in some species of *Cuscuta* is also flanked by one region that normally would contain an *ndh* gene (Funk et al. 2007, McNeal et al. 2007). However, a third inversion in *Cuscata* (Funk et al. 2007) is not flanked by *ndh* or other degraded genes, but it is noteworthy that one of the borders of this inversion is the same as we observe in *V. minimum*. Where we observe a major inversion of more than 24 kb in the LSC, a much shorter inversion spanning some 2 kb is found in *Cuscata*, but the border in the *trnT–psbD* region is shared.

In *V. minimum* the borders of the inverted region are highly AT-rich with microsatellite-like sequences, which may facilitate intramolecular recombination (Müller et al. 1999; Maréchal and Brisson 2010; Wicke et al. 2011). In general, the plastomes of *Viscum* have an increased number of repetitive DNA sequences and higher AT content compared with *Vitis*, but the same is not true for *Osyris* (table 2, fig. 3). Without data from more representatives of Santalales, in particular from autotrophs, we cannot conclude that the changes observed in *Viscum* are correlated with parasitism. In Orobanchaceae where several species were studied ranging from normal autotrophs to complete holoparasites, Wicke et al. (2013) showed a significant correlation between AT content and level of nutritional dependence. Structural changes (inversions and IR border modifications) and the amounts of repetitive DNA seemed to follow the same pattern. Among achlorophyllous, mycoheterotrophic plants, some orchids, and *Petrosavia* (Petrosaviaceae) also fit the pattern with a reduced plastome, gene loss, structural rearrangements, and modified IR borders, but not necessarily a high amount of repetitive DNA (Logacheva et al. 2014; Schelkunov et al. 2015). At the same time certain autotrophic plants have plastomes, which are highly rearranged (e.g., Cai et al. 2008; Haberle et al. 2008; Guisinger et al. 2011); hence, structural modification may be driven by several factors (Wicke et al. 2011).

A typical angiosperm plastome includes 113 genes: 79 protein-coding genes, 4 rRNA genes, and 30 tRNA genes (Wicke et al. 2011). In parasitic and mycoheterotrophic plants, numerous genes are pseudogenized or lost completely from the plastome (Funk et al. 2007; McNeal et al. 2007; Krause 2011; Logacheva et al. 2011, 2014; Wicke et al. 2011, 2013; Barrett and Davis 2012; Braukmann and Stefanović 2012; Braukmann et al. 2013; Li et al. 2013; Barrett et al. 2014; Lam et al. 2015; Schelkunov et al. 2015) and the most extreme reduction has been reached in holoparasitic *R. lagascae*, where no functional genes have been found (Molina et al. 2014). In *Cuscuta* and the Orobanchaceae, where several species have been investigated a clear correlation between gene loss/pseudogenization and degree of nutritional dependence on the host is observed (Funk et al. 2007; McNeal et al. 2007; Wicke et al. 2013).

The current data from Santalales confirm previous results. With a total number of functional genes ranging from 96 to 101 (supplementary table S3, Supplementary Material online) a reduction compared with normal angiosperm plastomes has occurred. Although the difference is not great, the facultative hemiparasite *Osyris* has retained more functional genes (101) than the obligate hemiparasitic species of *Viscum* (96–99 genes). This level of functional gene loss is comparable to the level observed in other hemiparasites and photosynthetic mycoheterotrophs (Funk et al. 2007; McNeal et al. 2007; Wicke et al. 2013; Barrett et al. 2014). Among the three species of *Viscum*, the largely endophytic habit *of V. minimum* has not led to further gene loss compared with the other species. *Viscum minimum* is even the species with most genes (99) retained. Although the exophytic tissue of *V. minimum* is reduced compared with other species of *Viscum*, it is green and clearly has photosynthetic capacity, thus it may not be surprising that the plastome gene content is not significantly different. Although measures of photosynthetic capacity have been made on *V. album* (Tuquet and Sallé 1996; Strong et al. 2000), similar measures have not been made in the other species. Below, we will discuss the loss and pseudogenization of individual genes observed in the Santalales in relation to other groups of angiosperms.

Most angiosperm plastomes include 11 genes coding for subunits of the plastid NAD(P)H-dehydrogenase complex involved in electron recycling around photosystem I (Krause 2011; Wicke et al. 2011). However, the genes may not be essential to plastid function as they are repeatedly lost or pseudogenized not only in all parasitic plants and mycoheterotrophs (Funk et al. 2007; McNeal et al. 2007; Krause 2011; Logacheva et al. 2011, 2014; Wicke et al. 2011, 2013; Barrett and Davis 2012; Braukmann and Stefanović 2012; Braukmann et al. 2013; Li et al. 2013; Barrett et al. 2014; Lam et al. 2015; Schelkunov et al. 2015) but also in some autotrophic plants, for example, species of Gnetales, Lentibulariaceae, Geraniaceae, Orchidaceae, and alismatids (Braukmann et al. 2009; Blazier et al. 2011; Iles et al. 2013; Wicke et al. 2014; Lin et al. 2015). Thus, the complete lack of functional *ndh* genes in *Osyris* and *Viscum* confirms previous findings, though the level of degradation may be surprisingly high. Retention of only one *ndh* pseudogene as we observe in *Viscum* has hitherto only been observed in holoparasites and achlorophyllous mycoheterotrophs (see Barrett et al. 2014). The facultative hemiparasite *Osyris*, which can survive without host contact, would be thought to be comparable to normal autotrophic plants, but with eight *ndh* pseudogenes and three genes lacking completely it adds to the evidence of general dispensability of the *ndh* gene complex.

Despite an increasing amount of available data from species of Orobanchaceae (Wolfe et al. 1992; Li et al. 2013; Wicke et al. 2013), *Cuscuta* (Funk et al. 2007; McNeal et al. 2007; Braukmann et al. 2013) and *Corallorhiza* Châtel. (Barrett et al. 2014), which has allowed for some insight into the evolutionary order of pseudogenization and gene loss, it is still not clear whether degradation of the *ndh* gene complex follows similar pathways in unrelated groups or whether a random mutation in any of the 11 genes can start the process. None of the plastomes investigated so far has just one degraded gene, but those with the smallest number suggest that possibly the first genes to be pseudogenized are *ndhD* and *ndhF* (compare Barrett et al. 2014; Wicke et al. 2014).

**Fig. 4.—:**
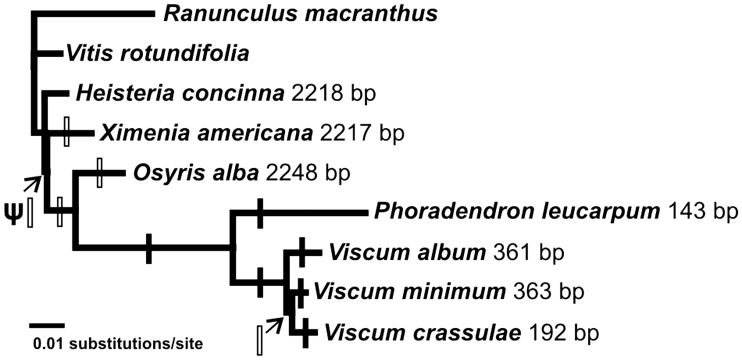
Phylogeny of the Santalales taxa studied here, based on maximum-likelihood analysis of the IR region, showing the progressive degeneration of the *ndhB* gene. Open bars symbolize minor indels (<15 bp), black bars symbolize major deletions (>50 bp). ψ indicates pseudogenization.

The most conserved *ndh* gene in Santalales is *ndhB*; probably not because it has a higher metabolic importance, but because of its position in the IR region, which has a stabilizing effect on sequences. Only very few plastomes of parasitic and mycoheterotrophic plants have lost *ndhB* completely (see Barrett et al. 2014). In Santalales, we can infer that the first steps in the degeneration process of *ndhB* are minor indels and substitutions followed by larger deletions (fig. 4).

The *infA* gene codes for translation initiator factor A protein (Wicke et al. 2011). It has been reported lost or pseudogenized in only a few parasitic plants, *Cuscuta* and *Conopholis* (Orobanchaceae), not in any mycoheterotrophic plants but in several autotrophic plants (Funk et al. 2007; McNeal et al. 2007; Wicke et al. 2011, 2013). Thus, despite that we observe pseudogene formation in *Osyris* and complete loss in the three species of *Viscum*, the changes may be unrelated to the hemiparasitic nature of these species.

A single ribosomal protein gene, *rpl33*, has been lost in all species of *Viscum*, but not in *Osyris*. The binding features of the L33 subunit are unknown, but the gene is only rarely lost (Wicke et al. 2011). It is known to be lost from the autotrophic *Phaseolus* L. (Fabaceae) and to be pseudogenized in the mycoheterotrophic orchid *Rhizanthella* R.S. Rogers (Delannoy et al. 2011; Wicke et al. 2011). Despite the apparent few losses, the gene does not seem to be essential and its loss may be unrelated to parasitism.

The product of the *matK* gene is believed to act as a splicing factor for plastid group IIA introns (Wicke et al. 2011). In general, angiosperm plastomes include seven group IIA introns, which are spliced by the *matK* product (Zoschke et al. 2010), and in some species of *Cuscuta* these introns have been lost together with a loss or pseudogenization of *matK* (Funk et al. 2007; McNeal et al. 2007; Wicke et al. 2011; Braukmann et al. 2013). The only other described cases of loss or pseudogenization of *matK* are from achlorophyllous, mycoheterotroph orchids: In *Neottia* Jacq. and *Epipogium* J.G.Gmel. ex Borkh the gene is lost, in *Rhizanthella* it is a pseudogene (Delannoy et al. 2011; Logacheva et al. 2011; Schelkunov et al. 2015). However, in all species at least some group IIA introns remain, and in *Rhizanthella* they are correctly spliced, suggesting that another gene was responsible for group IIA intron splicing in orchids (Delannoy et al. 2011; Logacheva et al. 2011). Although many orchids have been suggested to include *matK* pseudogenes, Barrett and Davis (2012) demonstrated that the plastome of the mycoheterotrophic but photosynthetic orchid *Corallorhiza* included a normal *matK* gene, where a pseudogene copy was located in either the mitochondrion or the nucleus. If the gene duplication has occurred basal to the orchids or it has occurred repeatedly, it is possible that some orchids have retained a functional *matK* gene in the plastome, whereas others have a functional copy in another genomic compartment provided that the product can be transferred into the plastids. Our observation of a *matK* pseudogene in *V. album*, coupled with presence of six of the seven *matK*-spliced groups IIA introns (the *trnV-UAC* gene been entirely missing), adds further evidence to the hypothesis that presence of a functional *matK* gene in the plastome is not essential for group IIA intron splicing. It is equally consistent with a hypothesis that a functional *matK* copy is located elsewhere and/or a hypothesis that an entirely different gene codes for the needed splicing factor.

The *ccsA* gene codes for a cytochrome C biogenesis protein, which mediates heme attachment to c-type cytochromes (Wicke et al. 2011). It is conserved among photosynthetic plants, but it is lost or pseudogenized in holoparasitic members of Orobanchaceae and *Cuscuta* and in achlorophyllous mycoheterotrophs (Delannoy et al. 2011; Logacheva et al. 2011, 2014; Wicke et al. 2011, 2013; Barrett and Davis 2012; Braukmann and Stefanović 2012; Braukmann et al. 2013; Li et al. 2013; Lam et al. 2015; Schelkunov et al. 2015). Thus, common to all reported cases of loss or pseudogenization is that they involved completely achlorophyllous plants; hence, a functional loss as we most likely observe in *V. album* is unexpected. In *Osyris* and *Vitis* the *ccsA* gene is 969 bp long, and the minor length reductions in *V. minimum* and *V. crassulae* to 948 and 954 bp, respectively, are unlikely to affect functionality. However, deletions interrupting the reading frame and leaving an open-reading frame of only 540 bp in *V. album* strongly suggest that the gene is nonfunctional.

Although typical angiosperm plastomes include 30 tRNA genes, it is not uncommon that species with no or reduced photosynthesis have lost a greater part of them (see, e.g., Lohan and Wolfe 1998; Wicke et al. 2011; Barrett et al. 2014) or perhaps even all (Molina et al. 2014). Thus, none of the two losses (*trnV-UAC* in all species and *trnG-UCC* in *V. album* and *V. crassulae*) that we observe in *Viscum* is unique and both involve tRNA genes which appear not to be essential as all species of *Viscum* still retain the *trnV-GAC* and *trnG-GCC* loci. The two loci, which are lost in *Viscum*, are preferentially lost also in other groups of parasitic and mycoheterotrophic plants (see Barrett et al. 2014), but in hemiparasitic species of *Arceuthobium* (Viscaceae) the *trnV-GAC* has been pseudogenized (Nickrent and García 2009). Unfortunately, is it not yet known whether these species instead have an intact *trnV-UAC* locus. Loss of both *trnV* genes has hitherto only been found in holoparasitic Orobanchaceae (Wolfe et al. 1992; Wicke et al. 2013) and some achlorophyllous mycoheterotrophs (Delannoy et al. 2011; Lam et al. 2015; Schelkunov et al. 2015).

Although complete gene loss can be regarded as a final step in evolution, prior steps may include accumulation of point mutations eventually leading to pseudogenization, and an increase in the ratio of nonsynonymous (d*N*) to synonymous (d*S*) substitutions, *ω*, is often used to demonstrate relaxed selection of genes (e.g., Krause 2011; Barrett et al. 2014; Wicke et al. 2014). Using the selection intensity parameter, *k*, we demonstrate that all the genes involved in photosynthesis show significantly relaxed selection in *Viscum* and *Osyris,* whereas the selection pattern for other gene groups seems more muddled with some being relaxed others seemingly being under increased selective pressure. Thus, the parasitic lifestyle has not consistently changed the plastome gene selection pressure. As both *Viscum* and *Osyris* appear as normal photosynthetic plants or with slightly reduced photosynthesis, only, it is surprising that particularly the photosynthesis genes seem to experience a relaxed selective pressure. Previous analyses of selection patterns in *Cuscuta* and *Corallorhiza* have found relaxation of selective pressure on photosynthesis genes, although relaxation may not be significant in *Cuscuta* (McNeal et al. 2007; Krause 2010, 2011; Barrett et al. 2014). However, in these genera with species with strongly reduced or even lacking photosynthetic capacity relaxation might be expected. More surprisingly, Wicke et al. (2014) discovered that also carnivorous plants from Lentibulariaceae had relaxed selection of plastid genes including some photosynthesis genes, but whether this can be correlated to photosynthesis, nutritional habit or some other physiological aspect of the carnivorous lifestyle remains unclear. Based partly on the lack of significance for relaxed selection pressure in *Cuscuta*, Krause (2010, 2011) suggested that evolution toward parasitism could happen stepwise rather than in a slow, continuous way. Although this may certainly be true, and pseudogenization and loss of *ndh* genes may illustrate such a domino effect, our data suggesting that initial modification of photosynthesis genes starts prior to loss or reduction of function may indicate that part of the evolution does happen in a slow, continuous manner.

Our present data do not allow for determining how early in the evolution of Santalales relaxation of selection pressure on photosynthesis genes takes place. The IR sequences available for some more representatives of the order do not include intact photosynthesis genes, and the only significant changes in selection that we can observe involve increases in selection pressure. The clearest change can be observed in the *ycf2* gene, which has an unknown function, and is associated with the evolutionary shift from facultative to obligate parasitism. Undeniably, the current taxon sampling is far too shallow to really understand the fundamental genetic changes and their potential correlation with types of parasitism and degrees of nutritional dependence of the host. However, future studies including a much broader range of representatives of all trophic levels in Santalales have the potential of providing a deep insight into the genetic consequences of parasitism.

## Supplementary Material

Supplementary tables S1–S3 are available at *Genome Biology and Evolution* online (http://www.gbe.oxfordjournals.org/).

Supplementary Data
