# Supplementary material for: Plastome Evolution in Hemiparasitic Mistletoes
Source: Genome Biol Evol. 2015 Aug 29;7(9):2520–32. doi: 10.1093/gbe/evv165 (PMC4607522; doi:10.1093/gbe/evv165)
Supplement: Supplementary Data [file supp_7_9_2520__index.html]

Plastome Evolution in Hemiparasitic Mistletoes — Plastome Evolution in Hemiparasitic Mistletoes — Supplementary Data 

# Plastome Evolution in Hemiparasitic Mistletoes

## Supplementary Data

files

- Supplementary Data - pdf file
