# Supplementary material for: Plastome Evolution in Hemiparasitic Mistletoes
Source: Genome Biol Evol. 2015 Aug 29;7(9):2520–32. doi: 10.1093/gbe/evv165 (PMC4607522; doi:10.1093/gbe/evv165)
Supplement: Supplementary Data [file supp_evv165_Supplementary_material.pdf]

Table S1. GenBank accession numbers of dicots with complete plastome sequences used for RELAX analyses.

GenBank acc.	Taxon
NC_008796*	*Ranunculus macranthus*
NC_009599*	*Buxus microphylla*
NC_026839	*Lonicera japonica*
NC_024663*	*Camellia reticulata*
NC_023790*	*Vitis rotundifolia*
NC_023112	*Helianthus tuberosus*
NC_023092*	*Liquidambra formosana*
NC_022810	*Aralia undulata*
NC_020098	*Tectona grandis*
NC_016068*	*Nicotiana undulata*
NC_010776	*Fagopyrum esculentum*
NC_008535*	*Coffea arabica*
NC_008359*	*Morus indica*
NC_008115*	*Eucalyptus globulus*

*) Sequences used for RELAX analyses of inverted repeat genes.

Table S2. Length in base pairs (bp) of intergenic regions shared by *Vitis* and four species of Santalales.

	Vitis	*Osyris*	*V. album*	*V. minimum*	*V. crassulae*
trnH-psbA	334	324	211	227	211
psbA-trnK	249	238	183	207	195
trnQ-psbK	346	354	245	211	248
psbK-psbI	406	382	372	408	404
psbI-trnS	142	139	92	95	110
trnR-atpA	198	173	220	226	164
atpA-atpF	65	51	76	80	74
atpF-atpH	493	163	368	307	129
atpH-atpI	895	1075	624	614	587
atpI-rps2	213	189	159	155	139
rps2-rpoC2	218	170	244	243	209
rpoC2-rpoC1	165	177	191	179	183
rpoC1-rpoB	31	26	26	26	26
rpoB-trnC	1084	836	454	445	467
trnC-petN	847	855	720	878	845
petN-psbM	1233	1146	412	419	423
psbM-trnD	1113	1170	409	501	404
trnD-trnY	445	426	459	414	401
trnY-trnE	54	59	64	62	68
trnE-trnT	921	802	524	601	582
psbC-trnS	246	226	228	233	228
trnS-psbZ	362	322	433	386	406
psbZ-trnG	627	232	213	229	216
trnG-trnfM	147	175	141	183	170
trnfM-rps14	167	148	147	147	141
rps14-psaB	131	141	136	134	138
psaB-psaA	25	25	47	52	32
psaA-ycf3	737	616	645	451	403
ycf3-trnS	678	981	499	545	486
trnS-rps4	272	302	324	310	322
rps4-trnT	301	376	188	172	196

trnT-trnL	975	479	570	637	562
trnL-trnF	321	376	268	332	309
trnM-atpE	235	213	278	273	267
atpB-rbcL	779	695	664	625	636
rbcL-accD	610	607	558	611	485
accD-psaI	760	682	180	159	175
psaI-ycf4	424	402	144	153	164
ycf4-cemA	858	715	494	450	364
cemA-petA	227	211	190	187	173
petA-psbJ	1074	514	389	402	374
psbJ-psbL	149	132	132	136	128
psbF-psbE	9	9	11	17	11
psbE-petL	1304	1241	861	883	769
petL-petG	179	167	167	162	162
petG-trnW	130	113	115	129	109
trnW-trnP	167	185	231	176	164
trnP-psaJ	383	377	367	351	304
rps18-rpl20	289	248	231	265	251
rpl20-rps12	789	777	800	793	759
rps12-clpP	142	130	145	163	154
clpP-psbB	443	417	1515	1538	514
psbB-psbT	187	185	175	180	186
psbT-psbN	52	65	52	66	74
psbN-psbH	84	84	100	103	95
psbH-petB	118	123	136	142	142
petB-petD	188	190	169	181	202
petD-rpoA	208	191	179	240	195
rpoA-rps11	80	65	66	69	59
rps11-rpl36	115	129	188	131	152
rps8-rpl14	169	142	136	132	136
rpl14-rpl16	125	121	105	138	132
rpl16-rps3	170	164	290	204	205
rps19-rpl2	69	111	55	115	55
rpl2-rpl23	18	18	13	13	13

rpl23-trnM	165	165	180	166	166
trnM-ycf2	88	88	88	88	67
ycf2-trnL	1007	1032	433	441	441
rps7-rps12	53	59	57	59	60
rps12-trnV	1717	1822	1782	1817	1309
trnV-16S	219	227	222	220	230
16S-trnI	300	300	321	316	307
trnI-trnA	63	64	62	61	62
trnA-23S	157	161	148	148	144
23S-4.5S	107	99	106	106	99
4.5S-5S	216	224	215	237	217
trnR-trnN	602	543	592	571	505
rpl32-trnL	1158	787	347	401	437
rps15-ycf1	302	391	133	124	123
ycf1-trnN	321	308	251	274	236
**TOTAL**	**31,450**	**28,847**	**23,965**	**24,325**	**21,490**
**Reduction**		**8%**	**24%**	**23%**	**32%**

Table S3. Gene content of plastomes of four species of Santalales. X = gene present and considered functional; ψ = pseudogene or fragment of gene present; – = gene not present.

	Osyris	*V. album*	*V. crassulae*	*V. minimum*
Protein coding genes				
psaA	X	X	X	X
psaB	X	X	X	X
psaC	X	X	X	X
psaI	X	X	X	X
psaJ	X	X	X	X
ycf3	X	X	X	X
ycf4	X	X	X	X
psbA	X	X	X	X
psbB	X	X	X	X
psbC	X	X	X	X
psbD	X	X	X	X
psbE	X	X	X	X
psbF	X	X	X	X
psbH	X	X	X	X
psbI	X	X	X	X
psbJ	X	X	X	X
psbK	X	X	X	X
psbL	X	X	X	X
psbM	X	X	X	X
psbN	X	X	X	X
psbT	X	X	X	X
psbZ	X	X	X	X
petA	X	X	X	X
petB	X	X	X	X
petD	X	X	X	X
petG	X	X	X	X
petL	X	X	X	X
petN	X	X	X	X
ndhA	ψ	–	–	–
ndhB	ψ	ψ	ψ	ψ
ndhC	ψ	–	–	–
ndhD	ψ	–	–	–
ndhE	ψ	–	–	–
ndhF	–	–	–	–
ndhG	ψ	–	–	–
ndhH	ψ	–	–	–
ndhI	–	–	–	–
ndhJ	–	–	–	–
ndhK	ψ	–	–	–
rbcL	X	X	X	X
atpA	X	X	X	X

atpB	X	X	X	X
atpE	X	X	X	X
atpF	X	X	X	X
atpH	X	X	X	X
atpI	X	X	X	X
rpoA	X	X	X	X
rpoB	X	X	X	X
rpoC1	X	X	X	X
rpoC2	X	X	X	X
matK	X	ψ	X	X
rps2	X	X	X	X
rps3	X	X	X	X
rps4	X	X	X	X
rps7	X	X	X	X
rps8	X	X	X	X
rps11	X	X	X	X
rps12	X	X	X	X
rps14	X	X	X	X
rps15	X	X	X	X
rps16	X	X	X	X
rps18	X	X	X	X
rps19	X	X	X	X
rpl2	X	X	X	X
rpl14	X	X	X	X
rpl16	X	X	X	X
rpl20	X	X	X	X
rpl22	X	X	X	X
rpl23	X	X	X	X
rpl32	X	X	X	X
rpl33	X	–	–	–
rpl36	X	X	X	X
infA	ψ	–	–	–
clpP	X	X	X	X
accD	X	X	X	X
ccsA	X	ψ	X	X
cemA	X	X	X	X
ycf1	X	X	X	X
ycf2	X	X	X	X
<u>Ribosomal genes</u>				
rrn16	X	X	X	X
rrn23	X	X	X	X

rrn5	X	X	X	X
rrn4.5	X	X	X	X
tRNA genes				
trnA–UGC	X	X	X	X
trnC–GCA	X	X	X	X
trnD–GUC	X	X	X	X
trnE–UUC	X	X	X	X
trnF–GAA	X	X	X	X
trnG–GCC	X	X	X	X
trnG–UCC	X	–	–	X
trnH–GUG	X	X	X	X
trnI–CAU	X	X	X	X
trnI–GAU	X	X	X	X
trnK–UUU	X	X	X	X
trnL–CAA	X	X	X	X
trnL–UAA	X	X	X	X
trnL–UAG	X	X	X	X
trnM–CAU	X	X	X	X
trnfM–CAU	X	X	X	X
trnN–GUU	X	X	X	X
trnP–UGG	X	X	X	X
trnQ–UUG	X	X	X	X
trnR–ACG	X	X	X	X
trnR–UCU	X	X	X	X
trnS–GCU	X	X	X	X
trnS–GGA	X	X	X	X
trnS–UGA	X	X	X	X
trnT–GGU	X	X	X	X
trnT–UGU	X	X	X	X
trnV–GAC	X	X	X	X
trnV–UAC	X	–	–	–
trnW–CCA	X	X	X	X
trnY–GUA	X	X	X	X
